# Data-driven leaf pruning based on weekly light integral: importance of dynamic defoliation strategy

**DOI:** 10.3389/fpls.2025.1651174

**Published:** 2025-09-25

**Authors:** Changhyeon Kim, Chieri Kubota

**Affiliations:** ^1^ Department of Plant Science and Landscape Architecture, University of Connecticut, Storrs, CT, United States; ^2^ Department of Horticulture and Crop Science, The Ohio State University, Columbus, OH, United States

**Keywords:** source and sink management, light compensation point, dynamic canopy management, high wire tomato production, controlled environment agriculture

## Abstract

**Introduction:**

High-wire tomato production requires labor-intensive tasks such as clipping, suckering, and leaf pruning. Leaf pruning is essential for managing a balance between vegetative and reproductive growth of plants. Commercial practices involve maintaining a certain number of leaves or no leaves below harvesting trusses. However, an optimum timing of leaf pruning for saving labor demand and improving crop performance is not well characterized.

**Method:**

Here, we introduce a data-driven leaf pruning method, in which lower leaves were removed when weekly light integral (WLI) below canopy fell below a pre-determined WLI based on the lowest leaf’s light compensation point (LCP). The number of leaves to prune at a time was three and a minimum pruning interval was one week. Additionally, we evaluated two ranges of photosynthetically active radiation (PAR): PAR (400 – 700 nm) and ePAR (400 – 750 nm) for monitoring WLI below the canopy. We compared the new leaf-pruning method based on WLI PAR (in Experiments 1 and 2) and WLI ePAR (only in Experiment 2) to the conventional leaf-pruning method, in which leaves below harvesting trusses were removed followed by harvesting (Control). For the evaluation, indeterminate tomato cultivar ‘Maxxiany’ was grown in a Venlo-style greenhouse (482 m^2^ and 7-m gutter height) at a density of 3 plants m^-2^.

**Results and discussion:**

Regardless of PAR range, the WLI-based pruning methods resulted in 35 - 42% fewer pruning events. The fewer pruning events were associated with the supplemental lighting use, leaving significantly more leaves per plant in the WLI-based pruning method than in Control. No significant differences were observed in the weekly increase in stem length, the stem diameter, and the cumulative yields between WLI-based pruning method and Control. However, WLI-based pruning method increased the total soluble solid contents of the harvested fruit. These findings suggest that: 1) Leaf pruning strategies should be adjusted based on light availability within the crop canopy, which is influenced by solar radiation and supplemental lighting, and 2) Monitoring WLI below canopy to determine leaf pruning timing is an effective method in lowering labor cost without reducing yield and fruit quality.

## 1 Introduction

High-wire tomato production in greenhouses is an ideal cropping system within controlled environment agriculture, enabling a consistent year-round production of fresh tomatoes. However, the production cost is significantly higher than field cultivation (Kubota et al., 2018). Various factors contribute to these higher expenditures, including energy for optimizing greenhouse environmental conditions, plant materials, fertilizers, and labor. Among these, labor is the most significant cost, accounting for up to 43% of the total production cost (Athearn et al., 2018; Testa et al., 2014).

The high-wire tomato production in greenhouses includes various tasks such as harvest, removal of lateral shoots, clipping main stems, truss pruning, lean and lowering, and leaf pruning (Kubota et al., 2018). Among them, leaf pruning plays an important role in maintaining an optimum number of leaves for canopy photosynthesis while removing leaves that are senescing or not contributing photosynthesis (sink). In other words, leaf pruning influences the available amount of carbohydrates for fruit production and thus yield (Adams et al., 2002; Kim et al., 2014). Additionally, leaf pruning can be used to manage a balance between vegetative and reproductive growth (McGraw et al., 2007), prevent disease outbreaks (Decognet et al., 2010; Silva et al., 2011), and improve water and fertilizer use efficiency (Adams et al., 2002).

To our knowledge, a common practice of leaf pruning by commercial greenhouse growers is to remove all leaves below the harvesting truss or second basal truss depending on the cultivar. Previously, several studies evaluated methods to optimize leaf pruning. Adams et al. (2002) compared two extreme degrees of leaf pruning: removing all leaves up to the second basal truss (an average of 16 leaves per plant) versus removing all leaves up to two trusses below the harvesting truss (an average of 28 leaves per plant). Leonardi et al. (2004) tested three degrees of leaf pruning: removing all leaves below the harvesting truss, the second basal truss, or the third basal truss. Kim et al. (2014) evaluated four pruning strategies: maintaining 14 leaves within the canopy, removing only one leaf above the harvesting truss, removing all leaves below the 7-week-old truss, and removing all leaves below the harvesting truss. None of these previous studies found a significant relationship between degrees of leaf pruning and yield. Moreover, their methods were not necessarily based on physiological evidence and may not be applicable to different growing conditions, especially under different light levels.

Alternatively, some studies have taken a more ecophysiological approach, using leaf area index (LAI; total leaf area divided by ground area) to evaluate the effects of leaf pruning on canopy light interception, crop growth, and yield (Heuvelink et al., 2004; Jo and Shin, 2020). While LAI-based leaf pruning may optimize canopy leaf area for improved yield, but this method has limited commercial applications because finding LAI requires destructive measurements of individual leaf areas, or complex canopy light transmission analysis using an expensive line quantum sensor, which may not be feasible for growers.

To simplify the decision for leaf pruning, we focused on light compensation point (LCP) of leaves as a decision-making criterion for leaf pruning. The LCP represents light intensity at which photosynthetic rate and respiration rate are equal (when net photosynthetic rate is 0 μmol m^-2^ s^-1^). Fully expanded leaves that intercept light intensity above the LCP have a positive net photosynthetic rate, likely functioning as sources (net exporters) of photoassimilates. Conversely, leaves exposed to light intensities below the LCP are likely sinks (net importers) of photoassimilates. Applying this principle to leaf pruning, our approach aims for all leaves within the canopy to function as sources by removing leaves receiving light intensity lower than the LCP.

Based on this concept, we developed a novel data-driven leaf pruning approach to determine the optimal timing for leaf pruning based on light availability below the canopy and predetermined leaf LCP. Since light intensity varies with various factors including time of day, season, weather conditions, shade-creating greenhouse components, and greenhouse orientations, relying on instantaneous light measurements could lead to overly aggressive pruning. To address this, we used weekly light integral (WLI) measured below the canopy to compare with a selected target (the weekly integral considering lowest leaf’s LCP) to evaluate the need of leaf punning. Furthermore, considering the unique light quality rich in far-red light in the canopy, we also evaluated the use of extended PAR (ePAR; 400–750 nm) spectrum for LCP measurements, following the previously reported study (Kim and Kubota, 2025).

We hypothesize that the novel data-driven leaf pruning approach can optimize leaf pruning timing in addition to enhancing tomato yield and quality, as it only retains leaves functioning as sources of photoassimilates. This study evaluated the effect of different leaf pruning methods (the data-driven methods *vs*. conventional method) by comparing growth parameters, yields, and fruit quality across two experimental trials from August 2023 to April 2024. Ultimately, we aim to demonstrate that our novel leaf pruning method optimizes yield, reduces labor input, and serves as a practical approach for commercial greenhouse tomato production.

## 2 Materials and methods

### 2.1 Methods

#### 2.1.1 The novel data-driven leaf pruning method

Our novel leaf pruning method essentially involves the following steps: (1) monitor light intensity below the lowest leaf by placing quantum sensors on selected substrate slabs, (2) compare the light intensity to a predetermined LCP of leaves to judge whether the leaf functions as a source or sink of photoassimilates, and (3) prune the lowest leaf as well as two additional leaves above it (three leaves in total) if the lowest leaf is considered as a sink due to the lower-than-LCP light intensity.

The problem with this approach is the changes in light intensity within or over days, which could make the same leaves as sink and source at a given time of observation. To address the issue, we used the weekly light integral (WLI) to consider whether the lowest leaves received a light integral that made the leaf as a net importer (sink) or an exporter (source) of photoassimilates when evaluated over a week.

We monitored WLIs on a daily basis (daily updated WLI) to decide whether leaf pruning was needed. In this approach, the threshold WLI (mol m^-2^ week^-1^) determining sink or source is given as the multiplication of LCP (mol m^-2^ s^-1^) by a total duration of photoperiods in a week (s week^-1^). Alternatively weekly averaged daytime light intensities can be used to compare with LCP; we use WLI in this study, as the wireless quantum sensors for PAR that we employed (DLI-500, Apogee instruments, Logan UT, USA) were designed to report daily light integral (DLI) and it is easy to find daily updated WLI. Additionally, the pruning strategy involves the removal of three leaves at a time, aligning with common practice in commercial high-wire tomato production and the morphology of tomato plant, a repeating pattern of three leaves, and a fruit truss to develop within the canopy. In order to avoid excessively frequent leaf pruning, a minimum interval between pruning events was applied as seven days.

In our approach, we considered the LCP of the bottom leaf to determine the threshold WLI recorded by a quantum sensor placed below the leaf. In this case, we needed to adjust the WLI, because the light intensity below the bottom leaf is lower than the actual light intensity reaching the leaf. Alternative approach could be placing the sensor at the height just above the bottom leaf; however we consider the approach less feasible as the height of the bottom leaf may vary depending on the leaf pruning and we did not want to create any obstacles for other growers tasks such as leaning and lowering the plants. For adjusting the WLI, we applied a predetermined correction factor to the WLI measured below the bottom leaf. This correction factor was also predetermined based on vertical profiles of photosynthetic photon flux density (PPFD) measured using a line quantum sensor (LI-191R, LI-COR, Lincoln, NE, USA) in a preliminary experiment (n = 192). The line quantum sensor was positioned horizontally within the canopy, with the sensing surface facing upward. Measurements were taken at multiple canopy depths, where depth was defined as the difference between the maximum height of the canopy and the height of each PFFD measurement. The measurements were conducted on 30 randomly selected plants, with 6–8 measurements per plant, as PPFD was recorded at 50 cm intervals, while canopy height varied. The relative light intensity at each measurement depth (PPFD at depth/PPFD above the canopy) was fitted to an exponential decay curve following the Lambert-Beer law: Relative light intensity = e^-k × depth^, where k is the light extinction coefficient (Nobel and Long, 1985).

From these vertical profiles in ‘Maxxiany’ cultivar, we obtained k of 1.54. The same experiment also showed that the difference in average vertical distance between the bottom leaf and the WLI measurement location was 0.07m. Using these values, the correction factor (1.11) was computed as the ratio between relative light intensities at the bottom leaf and those at 0.07 m below the bottom leaf (where the quantum sensor was placed). Specifically it was computed from the equation: e^-1.54×(z – 0.07)^/e^-1.54×(z)^ = e^1.54 × 0.07^ = 1.11 (where z represents the depth of the bottom leaf). This factor may vary under different growing conditions that alter leaf size and consequently, light extinction coefficient of the canopy. However, for simplification, we assumed that the same factor could be applied throughout the our experiments.

#### 2.1.2 Light compensation point of lower leaves

For finding LCP, prior to initiating the new pruning method, we measured leaf net photosynthetic rates of bottom leaves using a portable leaf photosynthesis measurement system (CIRAS-3, PP system, Amesbury, MA, USA) set at varied PPFD. The measurements (n = 8) were fitted into a common net photosynthesis (P_n_) model (P_n_ = A_max_ × (1 – exp(-QY_max_ × PPFD) - R_d_), where A_max_, QY_max_, and R_d_ are light-saturated gross photosynthetic rate, maximum quantum yield of CO_2_ assimilation, and dark respiration rate, respectively. The LCP was found as an x-axis intercept (PPFD giving zero P_n_) using the model. In the first experiment, measurement PPFD were 0, 15, 30, 60, 100, 200, 400 μmol m^-2^ s^-1^ and LCP was found as 32 ± 11 μmol m^-2^ s^-1^ (**Supplementary Figure S1**). Weekly light integral of LCP (WLI LCP) at the lowest leaf was then estimated as 11.3 mol m^-2^ week^-1^ for a given photoperiod (14h). In the second experiment, the LCP were determined as 27.8 µmol m^-2^ s^-1^ (400–700 nm) and 34.2 µmol m^-2^ s^-1^ (400–750 nm) similarly with varied light intensities (PAR: 0, 11.6, 27.6, 50.9, and 63.3 µmol m^-2^ s^-1^; ePAR: 0, 11.8, 34.6, 61.9, and 89.2 µmol m^-2^ s^-1^) as previously reported (Kim and Kubota, 2025). The WLI LCP of PAR and ePAR were 11.2 and 13.8 mol m^-2^ week^-1^, respectively, for the 16-h photoperiod. The LCP and WLI LCP were also assumed unchanged over time in the present experiment.

### 2.2 Experiments

#### 2.2.1 Experimental site and greenhouse systems

A Venlo-style greenhouse located at The Ohio State University Controlled Environment Agriculture Research Complex (Columbus, OH, USA) was used in this study. A north-end compartment (7 m gutter height; 21.7 m × 22.2 m floor area) covered with diffuse-type ethylene-tetrafluoroethylene film (F-Clean Diffused, AGC Green-Tech Co., Ltd., Tokyo, Japan) for the roof and single-layer glass for the side walls was utilized. Overall PAR transmission of this compartment was approximately 50%. Greenhouse environment was controlled by Priva Climate Computer (Priva BV, De Lier, The Netherlands) equipped with various systems including: roof ventilators (operated by CR63E/4 motor, Bonora, Cento, Italy), cooling fans (Slant-wall Exhaust Fan, ACME, Muskogee, OK, USA), evaporative cooling pads (AquaCool evaporative cooling system, FarmTek, South Windsor, CT, USA), heating pipes (StarFin, DuoFin, and SunFin heating systems, BioTherm, Cotati, CA, USA), vertical air circulation fans (Multifan V-FloFan, Vostermans Ventilation B.V., Venlo, The Netherlands), shade curtains (Harmony 2047 FR, Ludvig Svensson, Malmo, Sweden), misting system (OASIS 88, Koolfog, Thousand Palms, CA, USA), supplemental LED lighting (Arize Element L1000, General Electric, Boston, MA, USA; emitting 77% of its photons in the red light range (600–700 nm) and no far-red light (700–800 nm) (**Supplementary Figure S3**), CO_2_ generator (Johnson Gas Appliance Co., Hiawatha, IA, USA), and fertilizer injection system (Nutrijet; Priva BV, De Lier, The Netherlands). Climate control strategies and setpoints were selected using a climate control software, Priva Office Direct (Priva BV, De Lier, The Netherlands) to achieve target environmental conditions.

#### 2.2.2 Plant materials and growth conditions

Indeterminate cherry tomato ‘Maxxiany’ (Axia Vegetable Seeds, Naaldwijk, Netherlands) was used. We chose this cultivar as it is commercially grown in our region due to its high market price. However, as indeterminant tomato cultivars have the same repeated structure (one cluster and three leaves), we expect the outcome of the experiment will be useful for any indeterminant cultivars in high-wire tomato production systems. Seeds were sown into rockwool plugs (36 mm *L* × 36 mm *W* × 40 mm *H*; AO PLUG, Grodan, Roermond, The Netherlands) covered with vermiculite on March 8, 2023 (Experiment 1) and November 28, 2023 (Experiment 2). The seedlings with six true leaves were transplanted into rockwool cubes (cube size: 10 cm *L* × 10 cm *W* × 6.5 cm *H*; NG2.0, Grodan, Roermond, The Netherland). The seedlings grown in cubes were finally transplanted to rockwool slabs (slab size: 100 cm *L* × 20 cm *W* × 7.5 cm *H*; NG2.0, Grodan, Roermond, The Netherland), when the first truss was developed with open flowers. The seedlings were clipped to hooks with twine (Turbo Hook, Paskal, Ma’a lot, Israel) (Mega vine clip, Bato Plastics BV, Zevenbergen, The Netherlands) at the final transplanting. A total of seven hundred plants were planted in the compartment with a planting density of 3 plants m^-2^.

**Figure 1:**
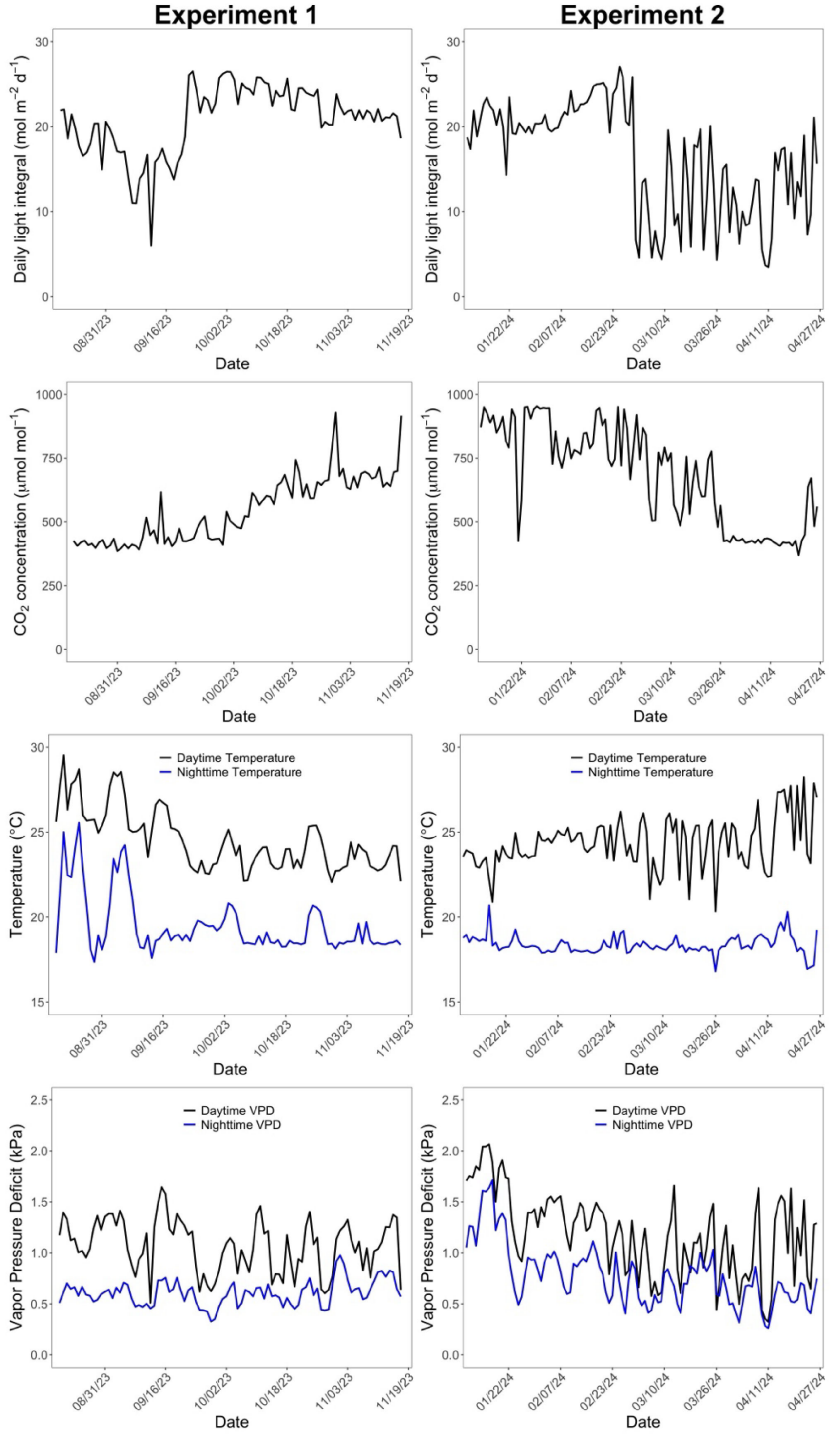
Changes of daily light integral, daytime CO_2_ concentration, day and night temperature, and day and night vapor pressure deficit over the cropping cycle. The first column represents the environmental conditions in the first experiment and the second column is from the second experiment.

The target day/night temperatures during the cultivation were 24/18°C. Target relative humidity (RH) during the day was 60%. Supplemental LED lighting (**Supplementary Figure S3**) was provided when outdoor solar radiation level was lower than 400 W m^-2^, after September 22, 2023 through February 29, 2024. The timing for starting (September) and ending (late February or early March) lighting followed the commercial greenhouse practices in Ohio. The daytime CO_2_ concentration was increased to the target CO_2_ concentration of 1000 μmol mol^-1^ when the greenhouse was not being ventilated. The average daily light integral (DLI) and daytime average CO_2_ concentration in the greenhouse were 20.7 ± 4.0 mol m^-2^ d^-1^ and 546 ± 126 μmol mol^-1^ in Experiment 1, and 17.0 ± 6.4 mol m^-2^ d^-1^ and 726 ± 180 μmol mol^-1^ in Experiment 2, respectively (**Figure 1**). Daytime temperature, nighttime temperature, daytime vapor pressure deficit, and nighttime vapor pressure deficit were 24.5 ± 1.8 °C, 19.5 ± 1.7 °C, 1.07 ± 0.26 kPa, 0.61 ± 0.12 kPa in Experiment 1 and 24.1 ± 1.2 °C, 18.3 ± 0.4 °C, 1.23 ± 0.38 kPa, 0.82 ± 0.30 kPa in Experiment 2 (**Figure 1**).

The tomato nutrient solutions were as published by Kroggel and Kubota (2018) with increasing concentration of micronutrient twice in Stage 3 recommended by commercial growers and the seed company (**Supplementary Table S1**). The three developmental stages included plants developing up to the 2^nd^ truss (Stage 1), from the 2^nd^ to the 5^th^ truss (Stage 2), and after the 5^th^ truss (Stage 3). Plants were fertigated at 100 mL per plant per irrigation event using a drip irrigation system with pressure-compensated emitters (1.9 L per hour; Netafim, Tel Aviv-Yafo, Israel). Daily irrigation volume per plant ranged from 0.6 to 4.0 L to achieve a daily drainage of approximately 30%, by adjusting irrigation frequency. The target drip EC was 1.8, 2.4, and 2.5 dS m^-1^, for Stages 1, 2, and 3, respectively, with a target pH of 6.0 for all three stages. The target drain EC was 2.5 to 3.5, 4.5 to 5.5, and 6.0 to 7.0 dS m^-1^, for Stages 1, 2, and 3, respectively, with a target pH of 6.0 to 7.0 for all three stages.

#### 2.2.3 Crop management

Typical high-wire tomato crop management procedures were applied. Namely, plant stems were clipped weekly to train the plants to grow vertically. All axillary shoots were removed to maintain only one stem per plant at least once a week. Truss pruning was conducted to retain 14 fruits per truss. Leaning and lowering the plants in rows were conducted biweekly only in Experiment 1 to maintain the canopy height at 3 m. In Experiment 2, plants were grown straight up to the maximum final height (4.3 m) without leaning and lowering, to allow for designing multiple pruning treatments in a row. Bumblebees (Standard Hive, Biobest, Westerlo, Belgium) were used to pollinate flowers by introducing a new hive once in six weeks. Beneficial insects to control white flies (Eretmix-System, Biobest, Westerlo, Belgium) were introduced biweekly as a preventative measure. Tomato trusses were harvested at least once a week when more than 90% of tomatoes in a truss were in the red stage, following the USDA Grade Standard for fresh tomatoes (USDA, 1991).

#### 2.2.4 Greenhouse experiments comparing leaf pruning methods

In Experiment 1, we compared two pruning treatments: (1) the new leaf pruning method considering WLI LCP (WLI PAR treatment) and (2) the conventional leaf pruning method (Control, removing all leaves below the harvesting trusses). The greenhouse compartment had two plots which were further divided into two subplots. The experimental design with the subplots is shown in **Supplementary Figure S2A**, where each subplot includes a plant row and each half of neighboring rows, assigned with the same pruning treatment (see color coding for each treatment in **Supplementary Figure S2A**). Within each subplot, we assigned two measurement blocks with 15 plants in the center of each subplot for plant growth and yield data collection. Each subplot had 140 plants as a replication of a pruning treatment. This was intended to standardize canopy structure around the measurement blocks and minimize light contamination between treatments. A wireless quantum sensor for PAR spectrum (DLI-500, Apogee instruments) was installed on a substrate slab below the lowest leaf at the center of each sampling section.

The new pruning method was introduced on August 19, 2023, when the plants had already developed a mature canopy structure. Until this date, the conventional leaf pruning method was applied for all plants in the greenhouse. At the beginning of the experiment, the WLI estimated at the lowest leaves in the WLI PAR treatment was far below the WLI LCP (11.3 mol m^-2^ week^-1^). Therefore, repeated leaf pruning was performed until the daily updated WLI below the canopy (measured at the slab surface) exceeded the target WLI (10.1 mol m^-2^ week^-1^). Afterwards, WLI LCP-based pruning was conducted with a minimum of seven days as the interval for the subsequent pruning as previously stated. The leaf pruning applied during the transition time in WLI PAR treatment was not counted in the comparison with Control. The experiment was terminated on November 17, 2023.

Experiment 2 was conducted with three treatments. In addition to the same two treatments examined in Experiment 1, a modified leaf pruning approach using extended PAR (ePAR; 400 to 750 nm) spectrum to measure WLI LCP (WLI ePAR treatment) was examined. The experimental design was a randomized complete block design with four blocks and three treatments (**Supplementary Figure S2B**). Each treatment within a block had 40 plants, all pruned using the same leaf pruning method. Among these, 10 plants within a designated sampling section were used for yield and crop data collection. A wireless quantum sensor for PAR (DLI-500, Apogee instruments) or ePAR spectrum (DLI-600, Apogee instruments) was installed on a substrate slab below the lowest leaf at the center of each sampling section.

As described earlier, the WLI LCP of PAR and ePAR were 11.2 and 13.8 mol m^-2^ week^-1^, respectively. The target WLI below the canopy at the slab surface was 10.1 and 12.4 mol m^-2^ week^-1^ for PAR and ePAR, respectively. The WLI PAR and WLI ePAR treatments were initiated immediately after transplanting onto the slabs on January 9^th^, 2024. As soon as daily-updated WLI recorded by the sensor fell short of PAR- or ePAR-based WLI, leaves in the treatments were removed. In Control, the initial leaf pruning was performed on February 7, 2024, to maintain a total of 18 leaves until the first harvest. After that, the leaf pruning in Control corresponded to the harvest, removing all leaves below the last fruiting truss. The experiment was terminated on April 26, 2024, when the plants reached the maximum height.

A power analysis was conducted to determine the minimum sample size. For both Experiment 1 and 2, the result indicated that four replications per each treatment is sufficient to detect large effects. In Experiment 1, two measurement blocks of 15 plants were sampled from a subplot containing 140 plants, whereas in Experiment 2, 10 plants were sampled from a subplot of 40 plants. Using a larger number of plants per subplot was necessary to minimize light contamination possibly neighboring plants subjected to different leaf pruning treatments. This approach also improves the precision of the data collected from each block. Therefore, we employed 4 replications per treatment in each experiment, while increasing the number of plants sampled within each replicate.

#### 2.2.5 Data collection

Weekly, one representative plant within each sampling section was used to measure stem length increase (cm), number of newly grown leaves (leaf length larger than 13cm), leaf length (measuring the 5^th^ leaf down from a leaf is larger length than 13cm in length; cm), stem diameter (measured at 15cm down from the shoot tip; mm), number of new flowering trusses, and distance between shoot tip and 1^st^ flowering truss. Additionally, number of leaves per plant was recorded weekly for all 15 plants in each sampling section in Experiment 1. In Experiment 2, only three representative plants per sampling section were used for this purpose.

All plants in each sampling section were used to collect marketable fruit yield (g), unmarketable fruit yield (g), number of trusses, number of marketable fruits, and number of unmarketable fruits weekly. These variables were used to calculate marketable yield (kg m^-2^) and percent marketable yield. Unmarketable fruits in these experiments were either unripe fruits or cracked fruits. Additionally, total soluble solid concentration (TSS, °Brix) in juice was measured every four weeks by using a handheld refractometer (PR-32α, ATAGO USA, Kirkland, WA) with randomly selected 10 fruits per sampling block.

At the end of Experiment 2, the newest fully developed leaf and the lowest (oldest) leaf (n = 4) were sampled for leaf mineral nutrient composition analysis at a commercial lab (JR Peters, Allentown, PA, USA). Drip and drain nutrient solution samples were analyzed prior to Experiment 1.

To assess canopy structures that affect vertical profiles in light intensity within the canopy, PPFD levels were recorded at various depths from the top of the canopy using a line quantum sensor (LI-191R, LI-COR, Lincoln, NE, USA). In Experiment 1, measurements were taken between June 15 and July 6, 2023, prior to the initiation of pruning treatments and on November 17, 2023, approximately three months after the initiation of leaf pruning treatments. Plants were not grown under supplemental light in June and July as the light use began after September 22, 2023. The supplemental lights were turned off when assessing the vertical light profile. Three plants within each measurement block were selected for measuring PPFD levels and the corresponding depths (**Supplementary Figure S2**). For each plant, measurements were taken at 50cm intervals along the stem, resulting in five to eight data points per plant depending on plant height. Thus, the number of measurements per treatment per measurement time ranged from 69 to 76 (3 replications × 2 blocks × 2 plots × varying depths). Changes in PPFD levels and the corresponding depths were fitted into an exponential decay curve (Relative light intensity = e^-^ *^k^* ^× depth^, where relative light intensity is the fraction of a PPFD level at a given depth to relative to the maximum PPFD level at the top of the canopy, and *k* is the light extinction coefficient) based on the Lambert-Beer law, following Nobel and Long (1985).

A similar methodology for assessing canopy structure was used in Experiment 2. Measurements were conducted on February 26 and March 29, 2024. Plants were grown under supplemental light in January and February but not in March (after February 29). The supplemental lights were turned off when assessing the vertical light profile. In Experiment 2, the number of PPFD measurements per plant was five, with varying intervals of depth ranging from 30 and 60cm. This resulted in 60 PPFD data points per treatment per date (3 replications × 4 blocks × 5 depths).

#### 2.2.6 Statistical analysis and data visualization

Statistical analysis and data visualization were conducted using functions and packages in R software version 4.2.2 (R Core team, Vienna, Austria). In Experiment 1, which included Control and WLI PAR, the “t.test” function was used to evaluate significance of the treatments on the number of leaf pruning events, cumulative yield, and total soluble solid. A two-way ANOVA using the “aov” function in R was performed to evaluate the effects of treatments and time on the changes in WLI below the canopy and leaf number. In Experiment 2, one-way ANOVA followed by mean separation using Tukey’s Honest Significant Difference test using “agricolae” package was conducted to evaluate the effects of treatments (Control, WLI PAR, and WLI ePAR) on the number of leaf pruning events, cumulative yield, and total soluble solids. A two-way ANOVA was then performed to assess the effects of treatment and time on the changes in WLI below the canopy and leaf number. For light extinction coefficient, a two-way ANOVA was conducted in both Experiment 1 and Experiment 2 to evaluate the effects of treatments and presence or absence of supplemental lighting. When treatments in each experiment did not show statistically significant difference, datapoints were combined for a t-test in Experiment 1 or an one-way ANOVA in Experiment 2. All figures in this manuscript were created using “ggplot2” and “dplyr” packages, with standard error bars.

## 3 Results

### 3.1 Crop headspace environmental conditions

Major environmental variables representing the greenhouse climate conditions in Experiments 1 and 2 are shown in **Figure 1**. Following the seasonal changes in outdoor solar radiation, DLI over the plants in Experiment 1 gradually declined over time for the first five weeks (ranged from 6.0 to 22.0 mol m^-2^ d^-1^). The use of supplemental lighting since September 22, 2023 substantially increased DLI over the plants, resulting in a range of 18.7 to 26.5 mol m^-2^ d^-1^. In contrast, in Experiment 2, DLI was maintained in a range of 14.3 - 27.1 mol m^-2^ d^-1^ during the first seven weeks and then declined due to turning off supplemental lighting after February 29, 2024 (ranged from 3.5 to 21.1 mol m^-2^ d^-1^). Daily fluctuations in DLI were greater without use of supplemental lighting than with lighting in both experiments.

Carbon dioxide concentration was elevated only when vents were closed in both experiments. As a result, CO_2_ concentrations higher than ambient levels were effectively achieved from September 22 to November 17, 2023 in Experiment 1 and January 9 to February 29, 2024 in Experiment 2, during which ventilations were limited for air temperature control with cooler climate conditions than in the rest of experimental period. Vapor pressure deficit of air did not show a clear pattern over time in Experiment 1, with 0.5 – 1.6 kPa during the day and 0.3 – 1.0 kPa during the night. In contrast, daytime VPD started with a relatively high value in Experiment 2 (0.8 to 2.1 kPa) during the first seven weeks, likely due to the smaller plant size at the start of the experiment.

### 3.2 WLI at the bottom of the canopy as affected by seasonal changes in incident light and canopy light interception

**Figure 2:**
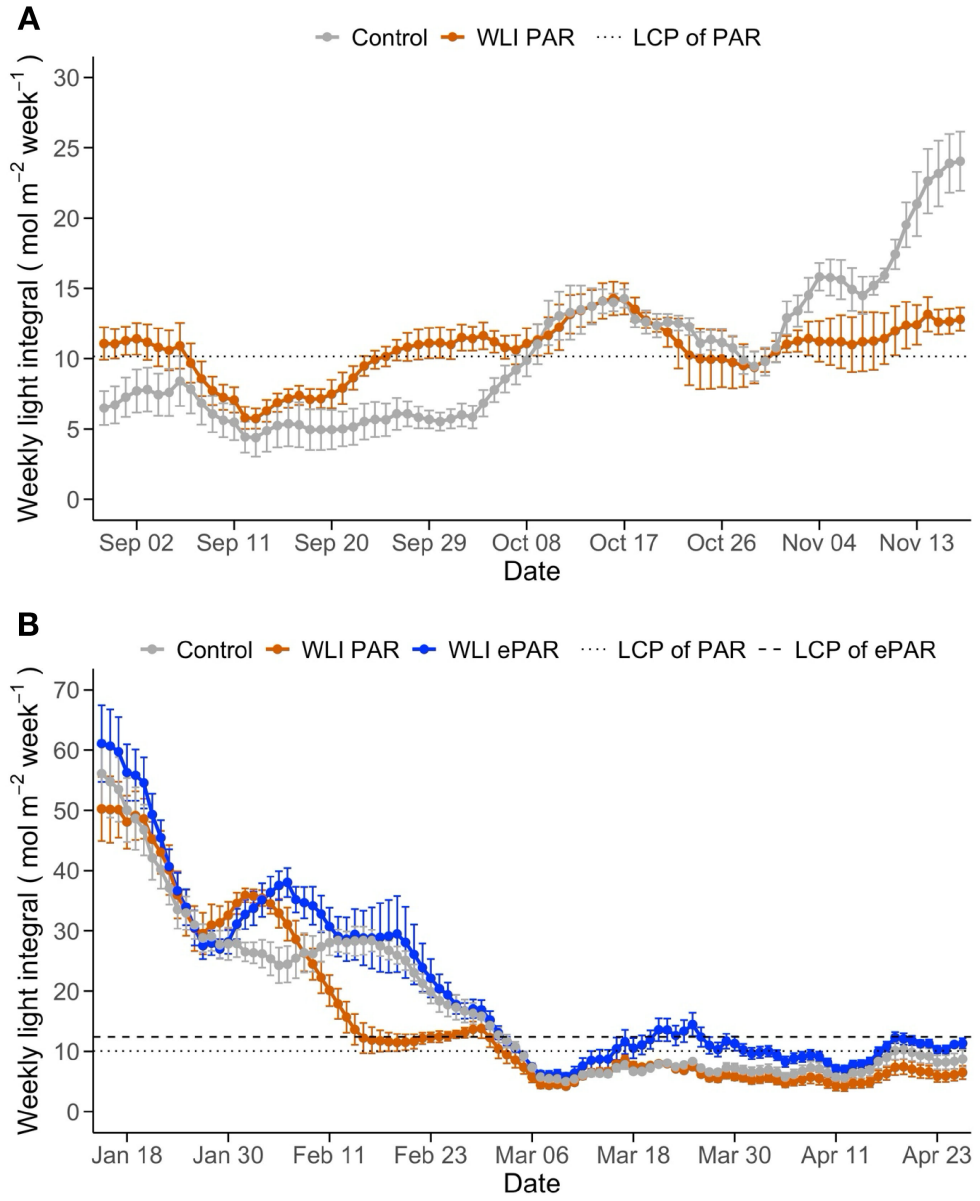
Changes of weekly light integral (WLI; 400–700 nm in **A** and 400–750 nm in **B**) below the canopy influenced by the leaf pruning treatments in Experiment 1 **(A)** and Experiment 2 **(B)**. Control, WLI PAR, and WLI ePAR represent the conventional leaf pruning method, the data-driven leaf pruning methods based on PAR (400–700 nm) and ePAR (400–750 nm), respectively. The dotted and dashed lines indicate the pruning threshold WLI at the bottom of canopy, determined based on the WLI light compensation point (LCP) of PAR and ePAR for the lowest leaves, respectively.

**Figure 3:**
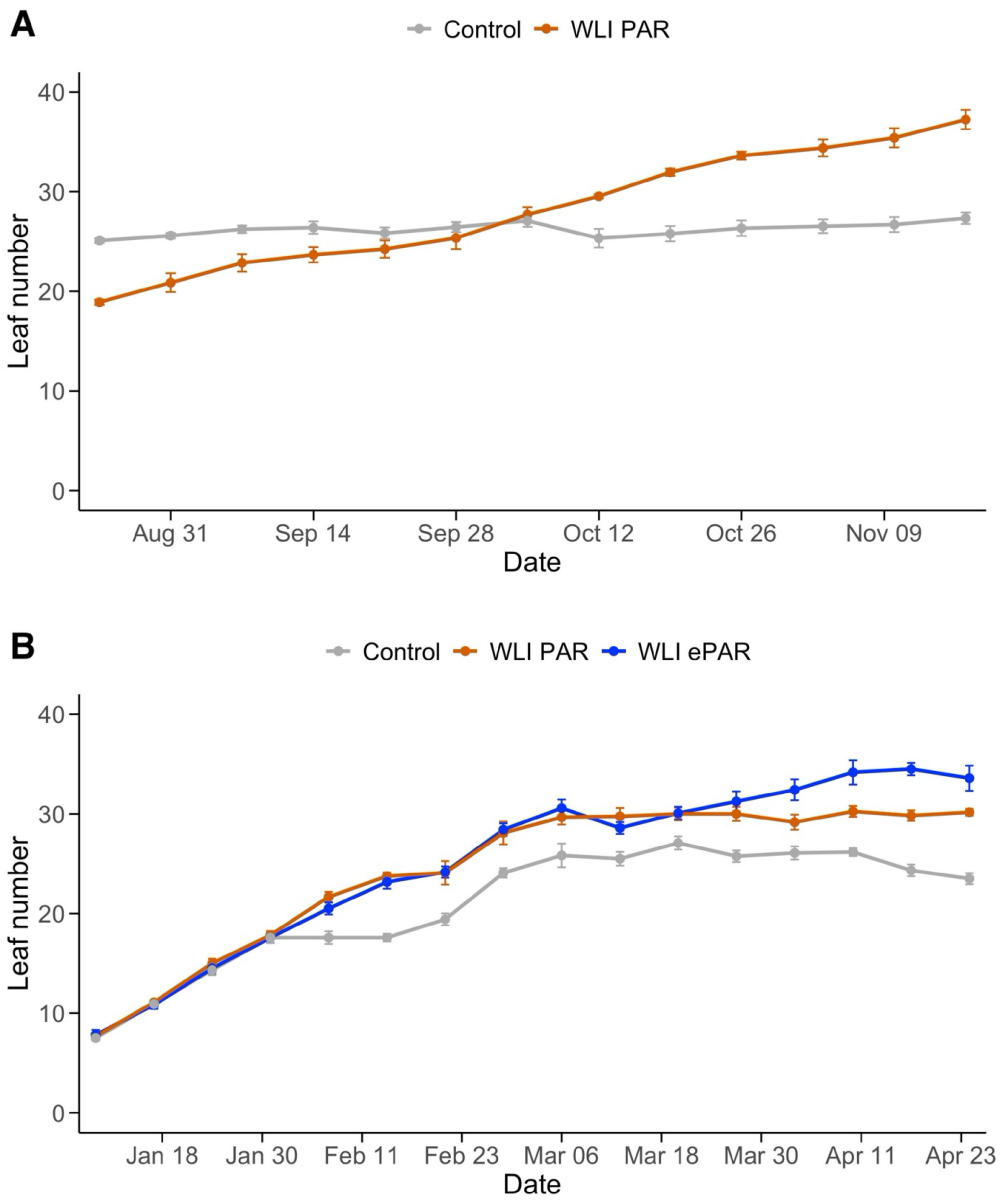
Changes of leaf number in Experiment 1 **(A)** and Experiment 2 **(B)**. Control, WLI PAR, and WLI ePAR represent the conventional leaf pruning method, the data-driven leaf pruning methods based on weekly light integral of PAR (400–700 nm) and ePAR (400–750 nm), respectively.

Changes of daily updated WLI below the canopy in response to the leaf pruning treatments are shown in **Figure 2**. In Experiment 1, WLI below the canopy (measured on the substrate slab at the bottom of canopy) in WLI PAR treatment ranged in 5.7 – 14.3 mol m^-2^ week^-1^, close to the target WLI 10.1mol m^-2^ week^-1^ on the slab (11.3 mol m^-2^ week^-1^ estimated over the lowest leaves) throughout the experiment. In contrast, WLI in Control gradually increased over time. Until September 22, 2023, when the use of supplemental lighting began, WLI in Control were lower than those in WLI PAR treatment (4.4 – 8.4 mol m^-2^ week^-1^ *vs*. 5.7 – 11.4 mol m^-2^ week^-1^) (**Figure 2A**). As soon as the start of supplemental lighting use, WLI in Control recorded a similar level (5.2 – 14.3 mol m^-2^ week^-1^) for about six weeks, and then consistently greater (14.5 – 24.1 mol m^-2^ week^-1^) compared with those in WLI PAR treatment (**Figure 2A**). These differences in WLI at the bottom of canopy are attributed mainly to the difference in number of leaves as affected by different leaf pruning methods and also changes in leaf size as affected by light intensity and quality, as discussed later. In Experiment 2, WLI recorded on the slabs at the bottom of the canopy declined over time (**Figure 2B**). The first pruning was applied in Control on February 6, 2024, four weeks after transplanting. The declining WLI was mainly due to the plant growth (increasing more leaves, **Figure 3B**) during the first four weeks. We also noticed small differences in WLI between experimental blocks, although no leaf pruning was applied. This was likely because the light measurements were affected by crop management practices such as adjusting vine clips to train vines vertically while minimizing the risk of leaves or trusses becoming entangled with heating pipes within the canopy.

In Control, the first pruning increased WLI, although overall declining trend remained. The timing of first pruning varied between four plots (**Supplementary Figure S2B**) for each treatment. In WLI PAR, the first leaf pruning occurred on February 15, 22, 29, and March 3 for the four plots (9 to 26 days after the first pruning in Control). In WLI ePAR, the first pruning was on February 18, March 2 and 5 for the four plots (12 to 28 days after the first pruning in Control, respectively). The late start of pruning in WLI PAR and WLI ePAR treatments compared with Control was due to relatively high WLI levels with the use of supplemental lighting. The average WLI in the WLI PAR treatment was closely aligned with the target WLI of PAR until around February 29, the end of supplemental lighting use (**Figure 2B**). WLI ePAR treatment showed higher WLI than its WLI target due to relatively large variation in WLI between experimental blocks. As Control plants were pruned weekly since the first pruning regardless of WLI, the WLI in Control became higher than those in WLI PAR treatment (**Figure 3B**). WLI ePAR treatment showed similar WLI as those in Control, due to the inclusion of far-red photons (700–750 nm) abundant below the canopy.

After February 29 (52 days after transplanting), no supplemental lighting was used, which reduced WLI in all treatments. The WLI in Control remained lower than WLI LCP (PAR, 11.2 mol m^-2^ week^-1^) for most of the time when no supplemental lighting was used. WLI below the canopy in WLI PAR and WLI ePAR treatments was at or greater than their target WLI only a few times toward the end of the experiment (2 and 6 times over a total of 57 days without lighting, respectively). The average WLI in WLI PAR and WLI ePAR treatments without supplemental lighting was 7.0 ± 2.0 and 11.2 ± 2.4 mol m^-2^ week^-1^, achieving only 62% and 81% of their target WLI values, respectively.

Changes in number of leaves are shown in **Figure 3**. In Experiment 1, Control plants were subject to regular weekly leaf pruning which resulted in a consistent number of leaves (26.2 ± 1.2 leaves per plant) throughout the experiment. WLI PAR treatment left an increasing number of leaves over time (from 18.9 ± 0.5 to 37.2 ± 1.9 leaves per plant) (**Figure 3A**), responding to the light available in the canopy. In Experiment 2, all treatments showed a similar number of leaves until January 31, 2024 (7.5 ± 0.7 to 17.8 ± 1.0) (**Figure 3B**). Since February 6, 2024, Control consistently had fewer leaves (approximately 3 to 9 leaves) compared to WLI PAR and WLI ePAR treatments. WLI PAR and WLI ePAR showed similar leaf numbers until March 27, 2024, with averages of 30.0 ± 1.3 and 31.2 ± 2.0, respectively. Such trend corresponds to their lower WLI levels than the target WLI thresholds upon the end of supplemental lighting use (**Figure 2B**). Meanwhile, as WLI ePAR treatment had a few days with higher WLI levels than the target WLI, resulting in a greater number of leaves since April 3, 2024. On the final day of Experiment 2, average number of leaves was 23.5 ± 1.1 for Control, 30.2 ± 0.7 for WLI PAR, and 33.6 ± 2.5 for WLI ePAR.

Number of leaf pruning events is summarized in **Table 1**. The total number of pruning events was 1.7 times greater in Control compared with WLI PAR treatment in Experiment 1. However, before the use of supplemental lighting, the number of pruning events was not significantly different in Experiment 1. The differences in the number of pruning events between Control and WLI PAR treatment were mostly shown during the period when supplemental lighting was in use (8.0 *vs*. 4.3 times), which resulted in significant difference in pruning events for the entire period (12.0 *vs*. 7.3 times). Experiment 2 showed that Control had 1.6 and 1.8 times greater total number of pruning events than WLI PAR and WLI ePAR treatments, respectively. The number of pruning events in Experiment 2 were significantly different among the treatments regardless of supplemental lighting use. However, the difference between Control and WLI treatments was more pronounced when supplemental lighting was used. Across both experiments, the data-driven leaf pruning method reduced pruning events by 35 – 43% compared to Control. In Experiment 2, the difference between WLI ePAR and WLI PAR treatments was not statistically significant. This likely indicates that inclusion of FR photons below the canopy in WLI ePAR treatment was not sufficient to lead to a significant difference in leaf pruning compared to WLI PAR treatment.

**Table 1:** Number of leaf pruning events throughout the experiments, with and without supplemental lighting (SL) (n = 4).

Experiment	Treatment	Total number of pruning events	Number of pruning events without SL	Number of pruning events with SL
Experiment 1	Control	12.0 ± 0.0	4.0 ± 0.0	8.0 ± 0.0
	WLI PAR	7.3 ± 1.8	3.0 ± 0.7	4.3 ± 1.1
	Significance	**	ns	**
Experiment 2	Control	14.0 ± 0.0 a	10.0 ± 0.0 a	4.0 ± 0.0 a
	WLI PAR	9.0 ± 0.8 b	8.0 ± 0.0 b	1.0 ± 0.8 b
	WLI ePAR	8.0 ± 0.8 b	7.8 ± 0.5 b	0.2 ± 0.5 b
	Significance	***	***	***

Statistical significance was assessed using a t-test for Experiment 1 and ANOVA for Experiment 2. *, **, and *** denote significant at *P* ≤ 0.05, 0.01, and 0.001, respectively. Different letters in Experiment 2 indicate significant differences among the treatments based on the ANOVA results.

### 3.3 Plant leaf nutrient concentration, growth, morphology and fruit yield

Mineral nutrient concentrations in upper and lower leaves under different leaf pruning treatments in Experiment 2 are shown in **Table 2**. Leaf senescence is typically marked by reductions in mobile macronutrients (N, P, K, and Mg) (Maillard et al., 2015), no significant declines were observed in the comparison. Instead, K concentrations of the bottom leaves were significantly higher in all treatments, contrary to the expected trend of senescing leaves. Additionally, Ca, an immobile nutrient that increases with senescence, was elevated in the lowest leaf of Control and WLI PAR treatment. These results suggest that the additional lower leaves left unpruned in the WLI PAR or WLI ePAR treatments were equally functional as those in Control rather than being in the process of senescing.

**Table 2:** Effects of leaf position on nutrient concentration under different leaf pruning treatments, including conventional leaf pruning (Control) and the data-driven leaf pruning methods based on weekly light integral of PAR (400–700 nm) and ePAR (400–750 nm) (n = 4).

Nutrient	Treatment	Top	Bottom	Significance
N (%)	Control	3.6 ± 0.23	3.8 ± 0.12	ns
	WLI PAR	3.9 ± 0.30	3.7 ± 0.11	ns
	WLI ePAR	3.7 ± 0.25	3.5 ± 0.41	ns
P (%)	Control	0.39 ± 0.012	0.51 ± 0.051	*
	WLI PAR	0.48 ± 0.059	0.55 ± 0.067	ns
	WLI ePAR	0.55 ± 0.043	0.59 ± 0.048	ns
K (%)	Control	4.8 ± 0.65	7.2 ± 0.36	**
	WLI PAR	4.5 ± 0.40	6.8 ± 0.11	***
	WLI ePAR	4.7 ± 0.38	6.1 ± 0.35	**
Mg (%)	Control	0.41 ± 0.017	0.47 ± 0.025	**
	WLI PAR	0.40 ± 0.053	0.50 ± 0.076	ns
	WLI ePAR	0.41 ± 0.039	0.54 ± 0.090	ns
Ca (%)	Control	2.3 ± 0.26	3.7 ± 0.25	***
	WLI PAR	2.0 ± 0.46	3.5 ± 0.35	**
	WLI ePAR	2.2 ± 0.58	3.0 ± 0.35	ns

Statistical significance (ns, *, **, ***) indicates non-significant, significant at *P* ≤ 0.05, 0.01, and 0.001, respectively, based on t-test comparing nutrient concentrations between top and bottom leaf positions within each treatment.

Weekly changes in stem diameter and distance between the first flowering truss and apical meristem did not show significant differences among the leaf pruning treatments in both experiments (data not shown). These values are often used for diagnosing the growth status of tomato plants as an indicator of balance between reproductive (also referred to as ‘generative’) and vegetative growth (da Costa, 2007; Kubota et al., 2018). Therefore, it seems that plants were in balance between reproductive and vegetative growth. This suggests that fewer pruning events in the data-driven leaf pruning did not influence plant growth status, even though harder (more aggressive) leaf pruning is a common practice to steer plant growth status from overly vegetative to more balanced status (McGraw et al., 2007).

**Figure 4:**
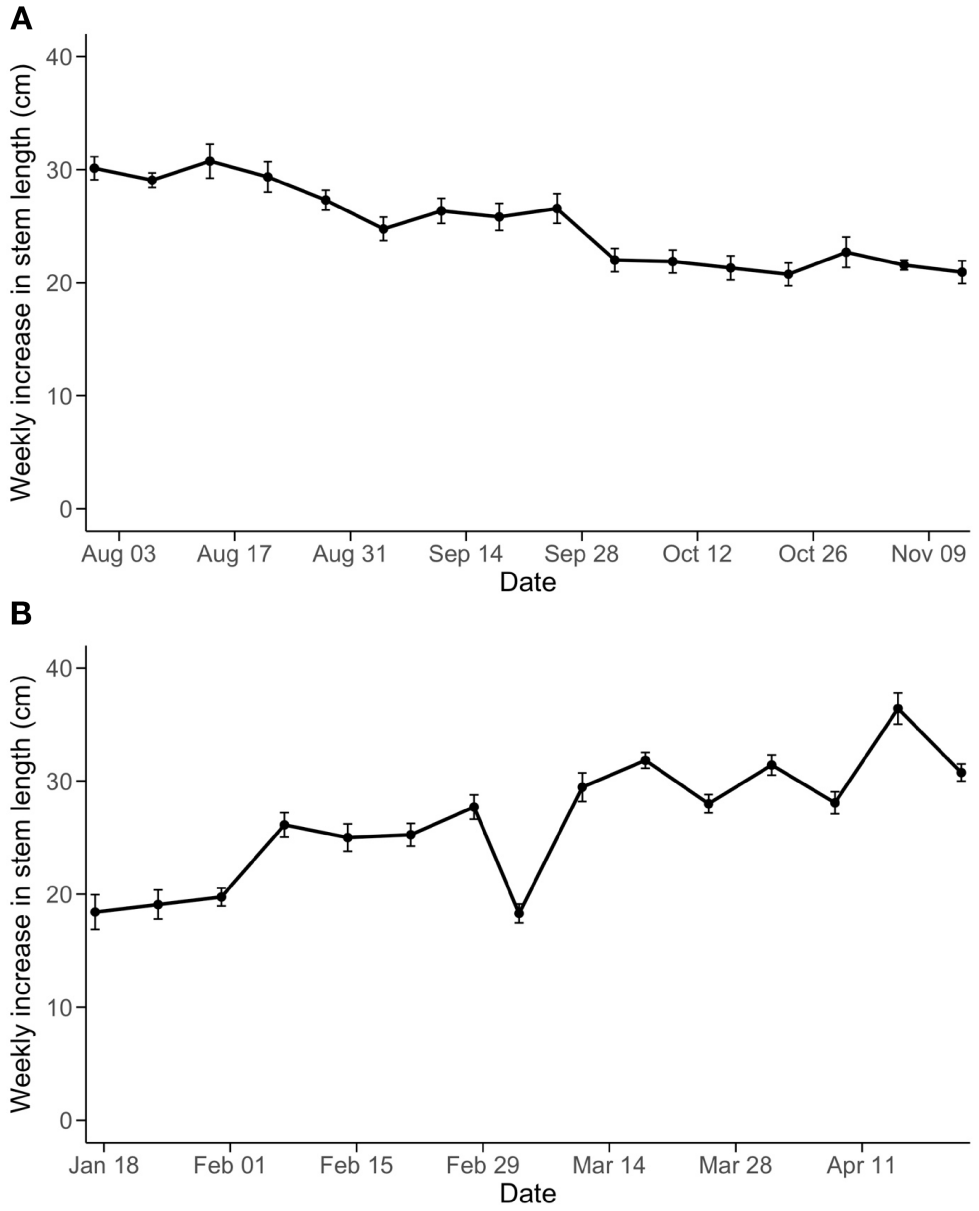
Changes of weekly increase in stem length in Experiment 1 **(A)** and Experiment 2 **(B)**. Since the leaf pruning treatments and their interaction with time were not statistically significant in both experiments, all data points at each week were combined to express temporal variation throughout the experiments.

**Figure 5:**
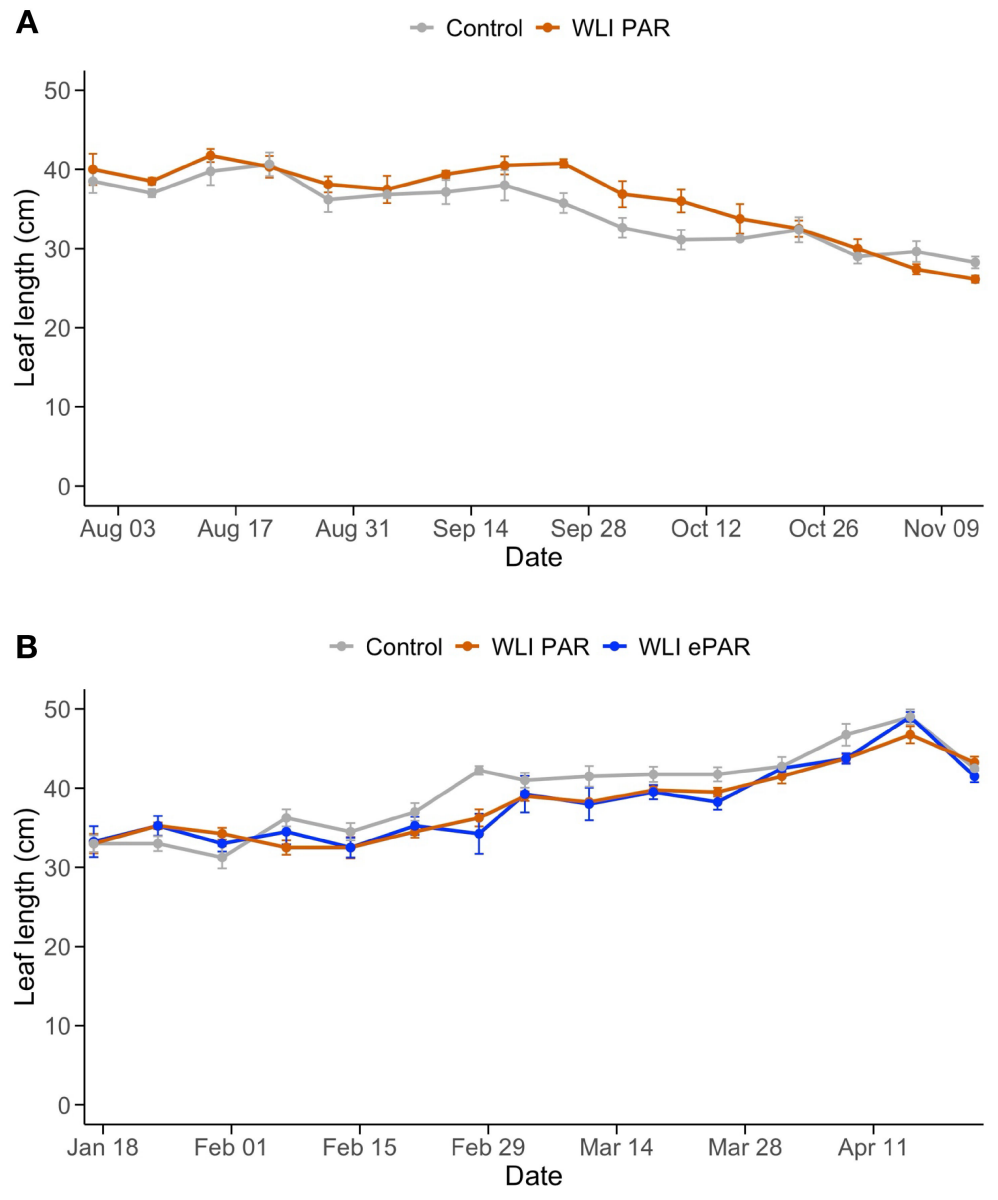
Changes of length of a fully expanded leaf in Experiment 1 **(A)** and Experiment 2 **(B)**. Control, WLI PAR, and WLI ePAR represent the conventional leaf pruning method, the data-driven leaf pruning methods based on weekly light integral of PAR (400–700 nm) and ePAR (400–750 nm), respectively.

Weekly increase in stem length (**Figure 4**) was not significantly affected by the leaf pruning treatments. Instead, changes in light intensity and light quality due to supplemental lighting use in the greenhouse likely influenced the weekly increase in stem length. Although leaf length was significantly influenced by the pruning treatments (*P*<0.01 in both experiments from the two-way ANOVA) (**Figure 5**), changes in leaf length also corresponded to the use of supplemental lighting. Specifically, the weekly increase in stem length and leaf length were in a declining trend in Experiment 1 (**Figures 4A**, **5A**) where overall light intensities were increased over time by use of supplemental lighting (**Figure 1**). Similarly, the increase in stem length and leaf length were in an increasing trend in Experiment 2 (**Figures 4B**, **5B**) where light intensities decreased over time. Supplemental lighting provided 64% and 68% of the total PAR during the periods when lights were used in Experiment 1 and 2, respectively.

Changes in stem elongation rate and leaf length affected the canopy structure, which then altered light transmission in the canopy. Our analyses of light within the canopy showed that supplemental lighting was the only factor significantly affecting the plant canopy structure. Significantly lower extinction coefficients (*k* values) were observed for plants grown with supplemental lighting use than without supplemental lighting (**Table 3**) in Experiment 1. In Experiment 1, k value under supplemental lighting was 58% lower than that without supplemental lighting. Lower *k* suggests that a larger fraction of incident light reaches to a deeper position within the canopy. However, Experiment 2 did not show the significant effect of supplemental lighting. This is likely associated with plant size, because the canopy was still developing when supplemental lighting was used during the first seven weeks of Experiment 2. Overall, reduction in *k* along with the increased light intensity by supplemental lighting contributed to greater WLI levels below the canopy and thus resulted in less frequent pruning in the data-driven leaf pruning (**Figure 2**, **Table 1**).

**Table 3:** Effect of supplemental lighting (SL) use on light extinction coefficient.

Experiment	Without SL	With SL	Significance
Experiment 1	1.53 ± 0.15	0.64 ± 0.08	***
Experiment 2	1.19 ± 0.35	1.11 ± 0.23	n.s.

Statistical significance was determined using two-way ANOVA. No significant effects of pruning treatment were observed without interaction with SL; therefore the data were pooled to analyze for SL effect, where n.s. and *** denote non-significant and significant at *P* ≤ 0.001, respectively.

The light extinction coefficient was calculated by fitting light measurements within canopy at various depths.

The WLI-based leaf pruning methods resulted in statistically similar cumulative yields in both experiments (**Table 4**). Marketable yield among the treatments in both experiments showed no statistical difference either (data not shown). Of interest, the TSS of fruit was greater in WLI PAR treatment than in Control, although the difference is small (0.4) in Experiment 1. A similar trend in TSS between WLI treatments and Control was shown in Experiment 2, although TSS was not significantly different between WLI PAR and Control. These results likely suggest that WLI PAR treatment optimized number of leaves within canopy for partitioning photoassimilates, contributing to fruit quality. However, optimized number of leaves in canopy in WLI PAR treatment did not result in increased fruit yield. In Experiment 2, WLI ePAR treatment showed similar yield and TSS as those in Control but lower TSS than in WLI PAR treatment (**Table 4**). Regardless, the difference between WLI PAR and WLI ePAR was small. Therefore, WLI PAR treatment appears to be a practical leaf pruning approach.

**Table 4:** Cumulative yield and total soluble solid concentration influenced by the leaf pruning treatments throughout the experiments (n = 4 per treatment).

Experiment	Treatment	Cumulative yield (kg m^-2^)	Total soluble solid (°Brix)
Experiment 1	Control	4.7 ± 0.1	9.8 ± 0.5
	WLI PAR	4.8 ± 0.4	10.2 ± 0.3
	Significance	ns	***
Experiment 2	Control	3.7 ± 0.2 a	9.1 ± 0.9 ab
	WLI PAR	3.6 ± 0.2 a	9.5 ± 0.9 a
	WLI ePAR	3.5 ± 0.2 a	8.9 ± 0.8 b
	Significance	ns	**

Yield was cumulated over 7 weeks in Experiment 1 and over 8 weeks in Experiment 2. Statistical significance was determined using a t-test for Experiment 1 and ANOVA for Experiment 2. ns, *, **, and *** denote non-significant or significant at *P* ≤ 0.05, 0.01, and 0.001, respectively. Different letters in Experiment 2 indicate significant differences among the treatments based on the ANOVA results.

## 4 Discussion

The novel data-driven leaf pruning method involves monitoring WLI levels below canopy and comparing them to WLI threshold below canopy for leaf pruning decision. The threshold WLI was selected so that the lowest leaves receive WLI LCP. The uniqueness of this approach lies in its decision-making process of leaf pruning based on light intensity relative to a photosynthetic parameter, LCP. Conventionally, optimizing crop management typically relies on decisions made by skilled growers, which can be challenging to standardize in a data-driven or echo physiological approach due to the complexity especially when crops, greenhouse systems, or environmental conditions are new to the growers. However, our data-driven leaf pruning approach simplifies by directly comparing WLI with target WLI thresholds to determine whether lower leaves in the canopy are still positively contributing to canopy photosynthesis. Based on our best knowledge, this is the first study that considered LCP for crop management decisions. Furthermore, this leaf pruning approach could be adopted for other crops and cropping systems that require leaf pruning, although optimization would need to account for difference in species and cultivars.

Pruning treatments did not show significant difference in plant growth and cumulative yield. However, the WLI-based leaf pruning method achieved significant reduction of leaf pruning events (**Table 1**), indicating labor savings of 35 – 43% under present experimental conditions. Since labor expenses account for up to 42% of the total production cost (Athearn et al., 2018; Testa et al., 2014), these labor savings for leaf pruning could substantially improve the profitability of high-wire tomato production. Additionally, the enhanced fruit quality in WLI PAR treatment with significantly higher TSS compared to Control (**Table 4**) was an additional advantage. Unbalanced growth status and more possibly senescing leaves at the lower side of canopy were concerns when we applied the WLI-based leaf pruning method; however, such issues were not observed in the present experiments (**Table 2**).

The advantage of using ePAR for WLI evaluations below the canopy is unclear based on the results obtained in this study. Specifically, WLI ePAR treatment showed significantly lower total soluble solid content compared to other treatments. Additionally, number of leaf pruning events in WLI ePAR treatment was not significantly different from WLI PAR treatment (**Table 1**). When plants were grown without supplemental lighting, there were times when WLI below the canopy was higher in WLI ePAR treatment than in Control or WLI PAR treatment. However, it is unclear how much of the far-red photons available for lower leaves actually contributed to photoassimilate production. Zhen et al. (2021) reported that far-red photons (700–750 nm) contributed equally to photosynthesis as red photons (600–700 nm), but only when the far-red photon flux density (PFD) does not exceed 30% of ePAR. However, in a dense tomato canopy under sunlight, far-red PFD can reach 44% of ePAR (Kim and Kubota, 2025). Therefore, although the treatment resulted in higher WLI for ePAR, these additional far-red photons are likely not contributing significantly to the increase of canopy photosynthetic rate. As PAR sensors are more widely available, it is more practical to use WLI PAR for commercial applications.

Our experiments demonstrated that leaf pruning strategies should be adjusted based on light availability as affected by season and supplemental light use. In both experiments, the WLI in Control was distinctly higher than that in the WLI PAR treatment when supplemental lighting was used (**Figure 2**). Under supplemental lighting, WLI PAR treatment resulted in fewer leaf pruning events and more leaves remaining within the canopy. This means that Control may have removed leaves that could have contributed to canopy photosynthesis. However, this did not negatively affect plant growth and fruit yields in the present experiments.

When supplemental lighting is a primary source of light, we observed shorter new stem growth and shorter leaves (**Figures 4**, **5**). These morphological changes are similar to plant responses to high light intensity and high red to far-red ratios provided by sole-source lighting (Wassenaar et al., 2022; Meijer et al., 2022; Zheng et al., 2023). The observed alterations in plant morphology are consistent with the fact that supplemental lighting contributed 64% and 68% of the total photons in Experiments 1 and 2 when supplemental lighting was in use, and the light fixtures for supplemental lighting did not include far-red photons (**Supplementary Figure S3**). While WLI-based leaf pruning approach responded to the changes in light environment inside the canopy as affected by the supplemental light use, Control did not have such a dynamic adjustment. However, in commercial greenhouses, growers may decide the position of removing leaves relative to the apparent change of canopy structure. Nevertheless, in our observation, it is rare to retain leaves below the ripening trusses in commercial greenhouses. Some growers believe that the ripening and color development of fruit are affected when fruits are shaded with neighboring leaves. Both ripening and lycopene synthesis are affected by temperature (Brandt et al., 2006), and lycopene synthesis is also affected by red light (Alba et al., 2000). Further investigation is needed to compare microclimates of ripening fruit under different pruning methods.

The WLI-based leaf pruning method may need further improvement to be more practical. While we did not examine in this experiment, plant density and cultivar-specific leaf traits (e.g., size and shape) can have similar influences on optimized leaf pruning. Although, it is not clear whether a minimum interval between each pruning event is needed. When the WLI below the canopy was low, leaves were pruned once a week because of the restriction of the minimum interval of leaf pruning in the methods examined in the two experiments. Specifically, under relatively low light conditions, leaf pruning by following the minimum interval did not help increase WLI values enough to reach the target WLI. This resulted in small or no differences in WLI between Control and the WLI-based leaf pruning (**Table 1**). However, given the limited impact of pruning methods on growth and crop yields, but the significant impact on labor reduction, users (growers) should decide the priority and in some cases (as in our Experiment 2), they can accept the lower WLI than target to save labor as a priority.

Additionally, the WLI-based pruning method may affect worker’s schedules and logistics in greenhouse crop management. Accommodating worker’s weekly schedules may result in conflict with the leaf pruning method. Commercial practices develop working schedules on a weekly basis. However, based on our historic leaf pruning events in our experiment, pruning events did not happen on a fixed day of the week but on any day as soon as WLI fell below the target WLI. The extra leaves left under ripening trusses in WLI-based pruning may make harvestable trusses less visible compared to removing all leaves below the harvesting trusses. Lastly, these additional remaining leaves may slow down the leaning and lowering process, as they get caught on canopy heating pipes or other structures on the gutters.

We admit that measuring LCP at commercial farms to implement this approach might not be practical. However, since our LCP measurements are within a similar range reported by other researchers (13.0 to 36 µmol m^-2^ s^-1^) (Gómez and Mitchell, 2016; Nederhoff and Vegter, 1994; Xiaoying et al., 2012), the WLI LCP values in this study could serve as a reference for implementing our data-driven leaf pruning method. Regardless of implementing our method, we strongly recommend monitoring light intensity both below and above the canopy. Monitoring light intensity below canopy provides valuable insights into the timing and frequency of leaf pruning, helping optimize crop management especially under high light conditions as shown in our experiments.

Our WLI-based leaf pruning method can be easily integrated into various instrumentation platforms to assist with leaf pruning timing or enable automated leaf pruning. The two key requirements for implementing this method are: 1) monitoring light intensity below the canopy and 2) comparing the daily updated WLI to a selected target WLI. By installing a quantum sensor below the canopy that is connected to the climate control system, it is relatively simple to add a rule that triggers an alarm when it is time for leaf pruning. In a separate experiment, we demonstrated a wireless PAR sensor-based alarm system for leaf pruning (Kim et al., unpublished). Additionally, this approach can be incorporated into automated leaf pruning machines, which might determine which leaves to remove based on the light intensities rather than the leaf position relative to the ripening trusses detected by machine vision.

In conclusion, we found that WLI-based leaf pruning method significantly reduced the number of leaf pruning events applied to high-wire cherry tomato crops in a greenhouse over two separate experiments. In addition, the newly developed method increased total soluble solid content of harvested fruits, although it did not significantly affect cumulative yield or growth parameters. These findings suggest that data-driven leaf pruning likely optimizes the number of leaves within the canopy, thus achieving a balance between source and sink within the canopy. This study also highlighted the differences in light intensities in the canopy when supplemental lighting was used. Our data-driven leaf pruning approach is a practical leaf pruning method applicable not only in the high-wire tomato industry, but also in other crops (e.g. cucumber) that require regular lower leaf pruning.

## Data Availability

The raw data supporting the conclusions of this article will be made available by the authors, without undue reservation.
