# Supplementary material for: Data-driven leaf pruning based on weekly light integral: importance of dynamic defoliation strategy
Source: Front Plant Sci. 2025 Sep 25;16:1651174. doi: 10.3389/fpls.2025.1651174 (PMC12509216; doi:10.3389/fpls.2025.1651174)
Supplement: Supplementary file 1 [file DataSheet1.docx]

Supplementary Material

# Supplementary Figures and Tables

## Supplementary Figures


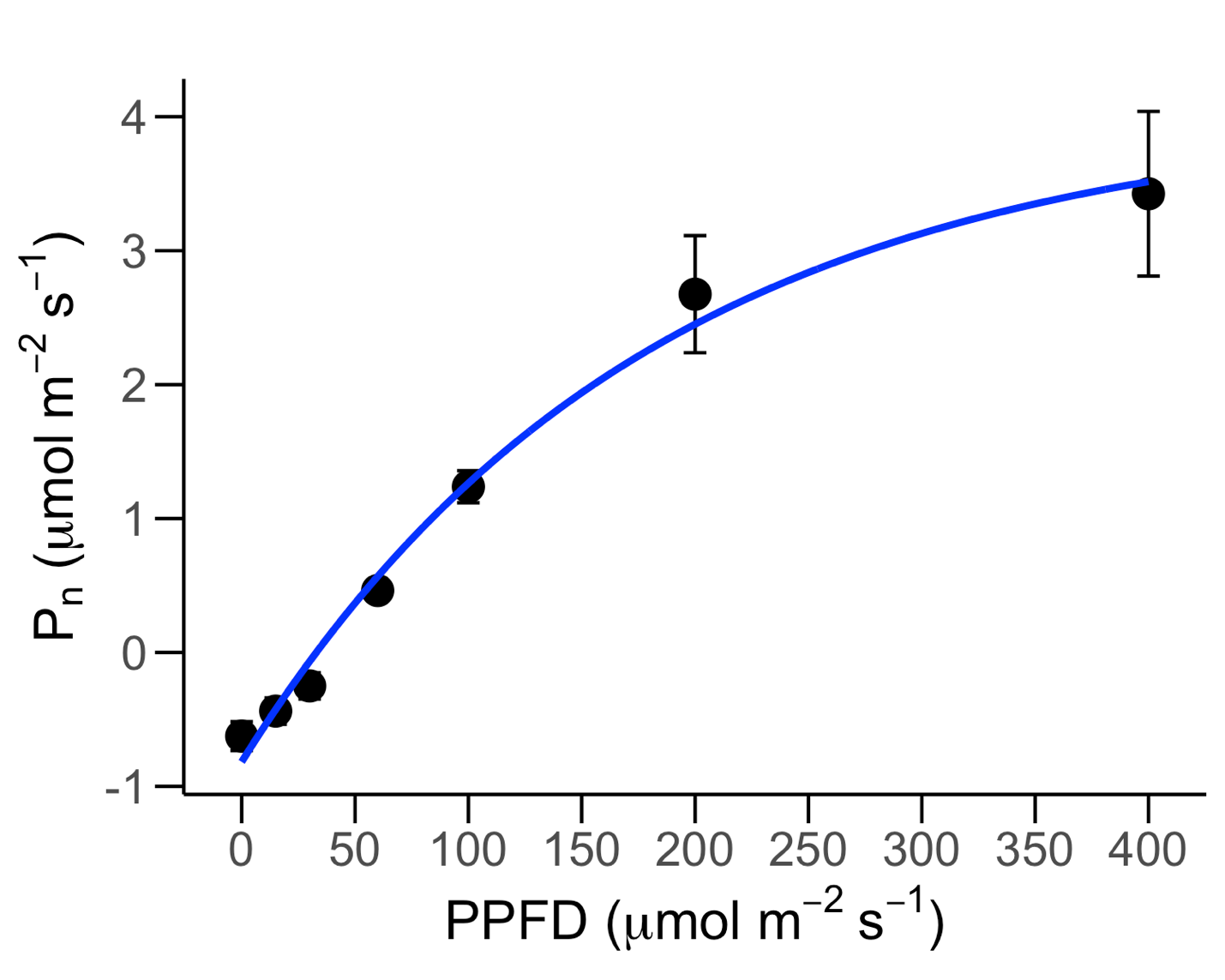


**Supplementary Figure 1**. Photosynthetic light response curve of leaves below the canopy (n = 8) under varying levels of photosynthetic photon flux density (PPFD). The blue line represents simulated net photosynthesis (P_n_) rates, derived from a non-linear regression analysis using a common photosynthesis equation (P_n_ = A_max_ × (1 – exp(-QY_max_ × PPFD) - R_d_), where A_max_, QY_max_, and R_d_ are light-saturated gross assimilation rate, maximum quantum yield of CO_2_ assimilation, and dark respiration rate, respectively. The A_max_, QY_max_, R_d_, and R^2^ were 5.4 μmol m^-2^ s^-1^, 0.0066 μmol mol^-1^, -0.83 μmol m^-2^ s^-1^, and 0.99, respectively.


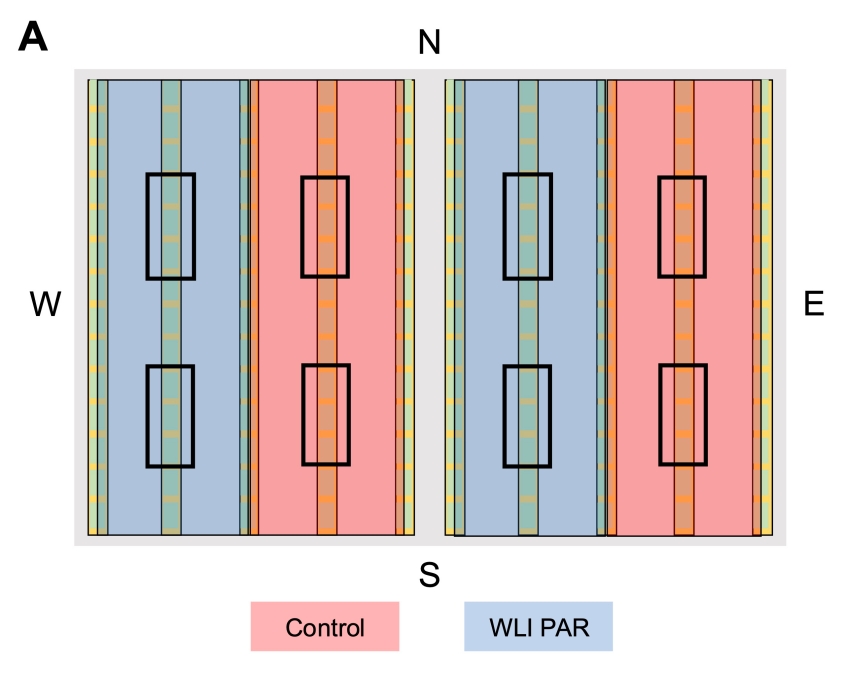

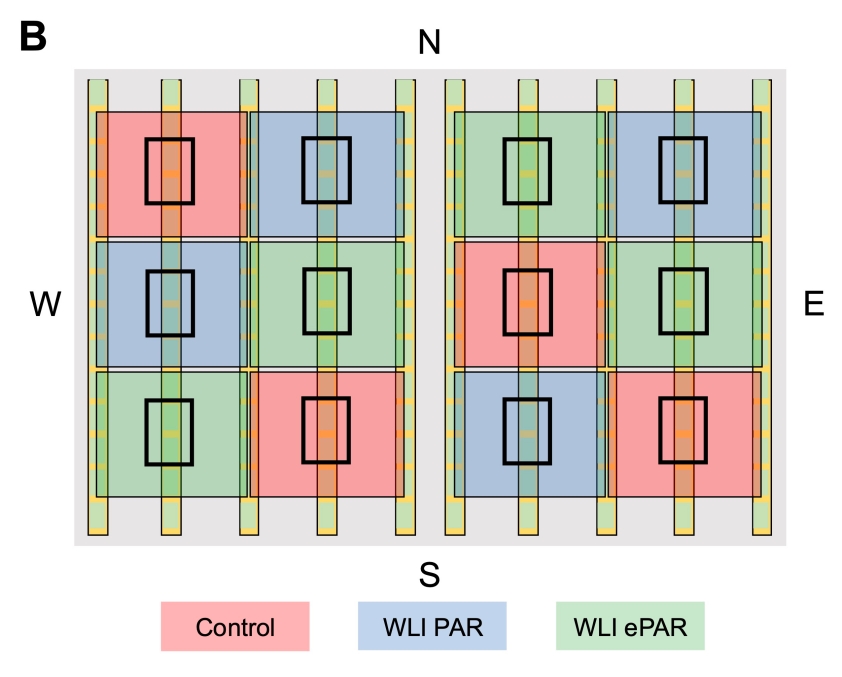


**Supplementary Figure 2**. Experimental plots of leaf pruning treatments in Experiment 1 (A) and Experiment 2 (B). “Control” (red) represents conventional leaf pruning, while “WLI PAR” (blue) and “WLI ePAR” (green) represent leaf pruning method based on weekly light integral (WLI) using photosynthetically active radiation spectrum (PAR; 400 to 700 nm) and extended PAR spectrum (ePAR; 400 to 750 nm), respectively. Boxes with thinner line represent the areas for each leaf pruning treatment, corresponding to the color of each treatment. The inner boxes with thicker line indicate areas designated for data collection. The narrow rectangular shapes are the locations of plant rows (double rows).


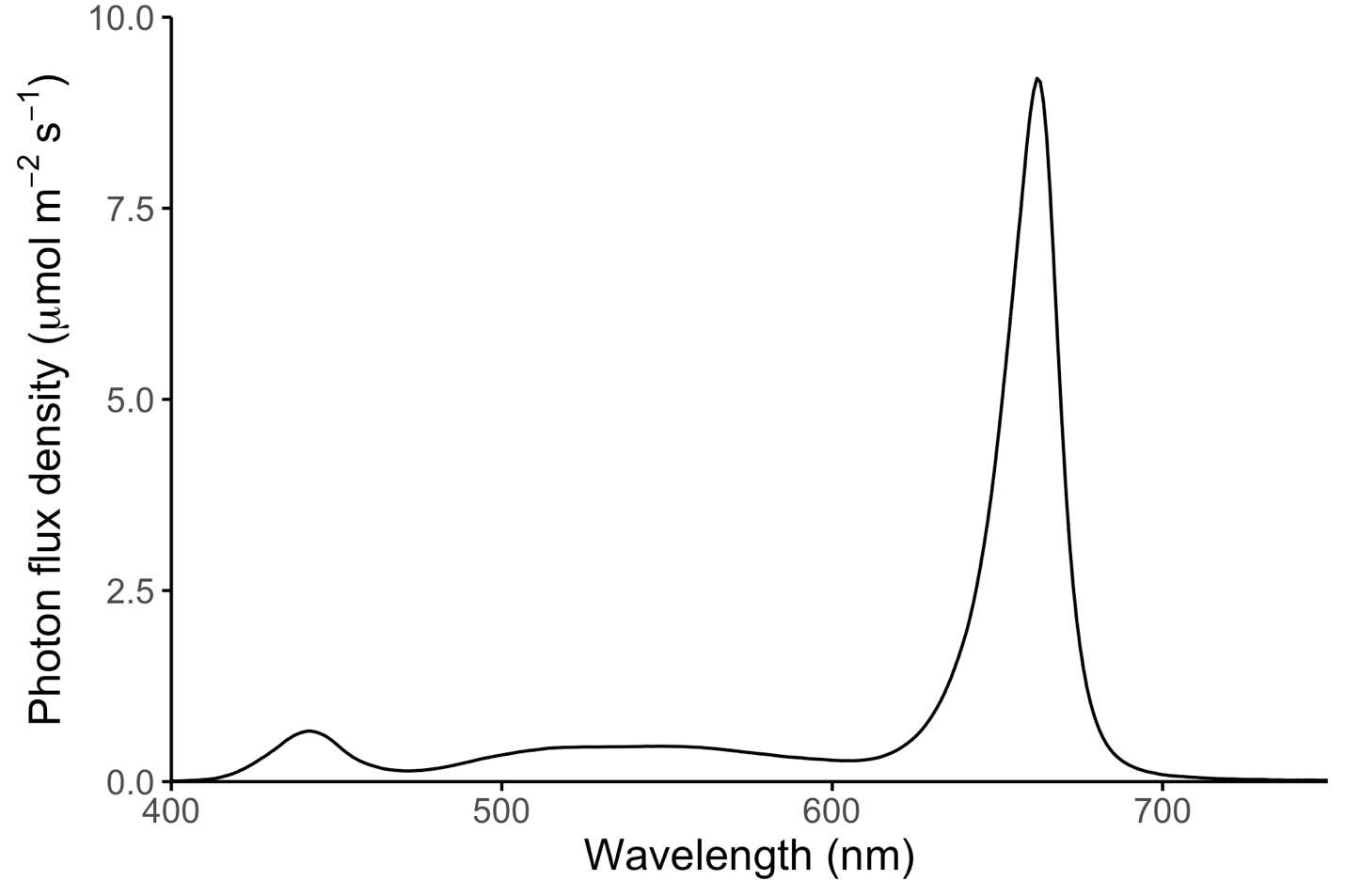


**Supplementary Figure 3**. Spectrum photon distribution of the light fixture for supplemental lighting.

## Supplementary Table

**Supplementary Table 1**. Nutrient element and its concentration in the multiple stages. The unit of values is mg L^-1^.

| Nutrient | Total N | NO_3_-N | NH_4_-N | P | K | Ca | Mg | *S* | *Cl* | Fe | B | Mn | Cu | Mo | Zn |
| --- | --- | --- | --- | --- | --- | --- | --- | --- | --- | --- | --- | --- | --- | --- | --- |
| Stage 1 | 90 | 90 | 0 | 47 | 144 | 144 | 60 | 116 | 89 | 2 | 0.34 | 0.55 | 0.05 | 0.05 | 0.33 |
| Stage 2 | 120 | 120 | 0 | 47 | 350 | 160 | 60 | 116 | 89 | 2 | 0.34 | 0.55 | 0.05 | 0.05 | 0.33 |
| Stage 3 | 240 | 240 | 0 | 47 | 350 | 200 | 60 | 116 | 89 | 2 | 0.68 | 1.1 | 0.1 | 0.1 | 0.66 |
